# Dysmenorrhœa

**Published:** 1859-09

**Authors:** 


### *Dysmenorrhœa.*

*Palliative treatment.*—The agent most serviceable of all, and that on which I cheifly rely for relieving the pain of dysmenorrhœa, is morphia in one or other of its forms. This generally acts better combined with some strong diffusible stimulant, as chloric either, which forms an admirable adjuvant to the action of the opium. The inhalation of chloroform, when the pain is severe, is often of the greatest use, for it is a singular fact that if you can bring the patient into the anæsthetic condition for only a short time at the very commencement of the attack, she will frequently remain free from pain whilst the discharge continues. There are only two local anodynes from which I have obtained any good results; these are carbonic aicd gas and the vapor of chloroform.

*Preventive Treatment.*—When dysmenorrhœa is kept up by a state of ovarian or uterine congestion or inflammation, we must try to reduce these states during the catamenial intervals, by repeated bleedings applied to the hæmorrhoidal vessels, by assiduous counter-irritation to the groins or sacrum, and by all the other usual internal remedies employed against local inflamation and congestion of other isolated organs of the body. If chiefly neuralgic in character, alteratives, and especally mineral tonics, good diet and regimen, and whatever tends to raise the standard of health, will, best cure the neuralgia.

For the gouty and rheumatic forms of the disease, useful results will be found from the administration of colchicum and guaiacum, either alone or in combination with alkalies. Bromide of potassium possesses a sedative action on the sexual organs possessed by no other drug in the pharmacopœia, and in many cases by its employment, you may succed in warding off attacks of dysmenorrhœa.

In the treatment of membranous dysmenorrhœa, besides the other remedies usually employed, powdered nitrate of silver may be applied to the uterine cavity by means of an instrument contrived for the purpose.—*Braithwaite.*

